# Ovarian steroid cell tumor in a teenager masquerading as polycystic ovary syndrome: a case report and literature review

**DOI:** 10.1186/s13048-025-01697-0

**Published:** 2025-06-02

**Authors:** Zhongyi Zhu, Risna Begam Mohammed Nazar, Xuan Zuo, Kaixuan Yang, Ting Wu, Yijing Zhang, Chenyan He, Kehui Xu, Yuedong He, Lin Li

**Affiliations:** 1 Department of Obstetrics and Gynecology, West China Second University Hospital, Sichuan University, 20 Ren Min Nan Lu, Chengdu, Sichuan 610041 P.R. China; 2 Key Laboratory of Birth Defects and Related Diseases of Women and Children (Sichuan University), Ministry of Education, Sichuan University, Chengdu, 610041 China; 3 Department of Pathology, West China Second University Hospital, Sichuan University, Chengdu, Sichuan 610041 P.R. China; 4 Sichuan Normal University, 5 Jing’an Rd, Jinjiang District, Chengdu, Sichuan 610021 PR China; 5 Department of Ultrasonic Medicine, West China Second University Hospital, Sichuan University, Chengdu, Sichuan 610041 P.R. China

**Keywords:** Ovarian steroid cell tumor, Polycystic ovary syndrome, Misdiagnosis, Hyperandrogenism, Rare ovarian tumors, Case report

## Abstract

**Background:**

Adolescent patients presenting with hyperandrogenic symptoms often receive a diagnosis of polycystic ovary syndrome (PCOS). However, accurate diagnosis is crucial because the symptoms of rare conditions, such as ovarian steroid cell tumor (OSCT), can mimic PCOS.

**Case summary:**

An 18-year-old female presented with irregular menstrual cycles, hyperandrogenic symptoms, and obesity. Despite standard treatments for PCOS, symptoms persisted. Exploratory laparoscopic examination revealed an OSCT. Thorough hormonal profiling, imaging, and histopathological analysis confirmed the diagnosis.

**Literature review:**

All OSCT and PCOS with their synonyms were searched in Pubmed on March 1, 2025. After limited the topic to tittle/abstract and then screened manually, only one report was found to present similar OSCT-NOS case with the case we report here.

**Conclusion:**

This case highlights the need for accurate diagnosis, early evaluation, and timely intervention in adolescents with hyperandrogenism to manage rare conditions like OSCT.

**Core tip:**

Adolescent females with hyperandrogenic symptoms are often diagnosed with polycystic ovary syndrome (PCOS). However, the symptoms of rare conditions like ovarian steroid cell tumors can mimic symptoms of PCOS. This case highlights the importance of an accurate diagnosis and a thorough evaluation through hormonal profiling, imaging, and exploratory laparoscopy. If the standard PCOS treatments are unsuccessful, then alternative diagnoses such as ovarian steroid cell tumor should be considered. Early identification and appropriate management are critical for satisfactory patient outcomes and emphasize the need for heightened awareness of rare conditions that present similarly to PCOS in adolescents.

## Background

Polycystic ovary syndrome (PCOS) is one of the most common endocrine and gynecological disorders in females [1]. PCOS affects 5-15% of females of reproductive age, and diagnostic variability is influenced by the diagnostic criteria used and ethnic background [2]. PCOS is a complex condition with a broad spectrum of clinical features. It is often associated with menstrual irregularities, hyperandrogenism, enlarged dysfunctional ovaries, insulin resistance, obesity, and reproductive challenges [3]. PCOS is often considered as the diagnosis before rarer diagnoses due to the similarity of symptoms in these diseases. This results in challenges for clinicians in obtaining accurate identification of the disease.

Ovarian steroid cell tumors (OSCTs) are very rare ovarian sex cord stromal tumors that have symptoms similar to the symptoms of PCOS. In a study conducted at the Perking Union Medical College Hospital of 7301 patients with ovarian tumors, only 14 cases (0.19%) were diagnosed as OSCT. OSCTs represent less than 0.1% of all ovarian neoplasms [4], which underscores the necessity for accurate differentiation. However, due to the rarity of OSCT, it is often and easily misdiagnosed. This case report and literature review highlights the complexities of accurately diagnosing an OSCT by distinguishing it from PCOS.

## Case presentation

### Chief complaints

An 18-year-old female presented with menstrual irregularity and progressive hirsutism that began at puberty.

### History of present illness

The patient experienced an 8-year history of irregular menstrual cycles and a 6-year history of hyperandrogenism, which worsened over time. The patient’s recent medical and menstruation details are presented in Table 1. She had been treated with DIANE-35 for 5 months to manage symptoms. However, there was no improvement in the hyperandrogenism during this period, and the medication was discontinued.

**Table 1:** Recent medical and menstruation history of the patient

Date	Type of visit	Reason for visit
April 29, 2022	Outpatient	Last menstrual period occurred 2 years prior
June 15, 2022	N/A	Menstruation occurred due to withdrawal bleeding induced by taking DIANE-35
June 20, 2022	Outpatient	Surgery recommended to patient
November 21, 2022	Outpatient	Patient took DIANE-35 for 5 months and discontinued in October 2022; last menstrual period was on November 1, 2022
February 2, 2023	N/A	Menstruation occurred
February 10, 2023	Surgical	Laparoscopic mass resection was performed
April 3, 2023	Postoperative outpatient	No menstruation occurred post-surgery
November 15, 2023	Outpatient	Last menstrual period was in August 2023

N/A: Not available

### History of past illness

The patient’s medical history revealed no significant prior illnesses nor surgical interventions except for the ongoing menstrual irregularities and hyperandrogenism.

### Personal and family history

The patient had no significant personal history of chronic illness, surgeries, or hospitalizations before the current issue. Family history was non-contributory, with no known genetic or endocrine disorders in her immediate family.

### Physical examination

**Fig. 1:**
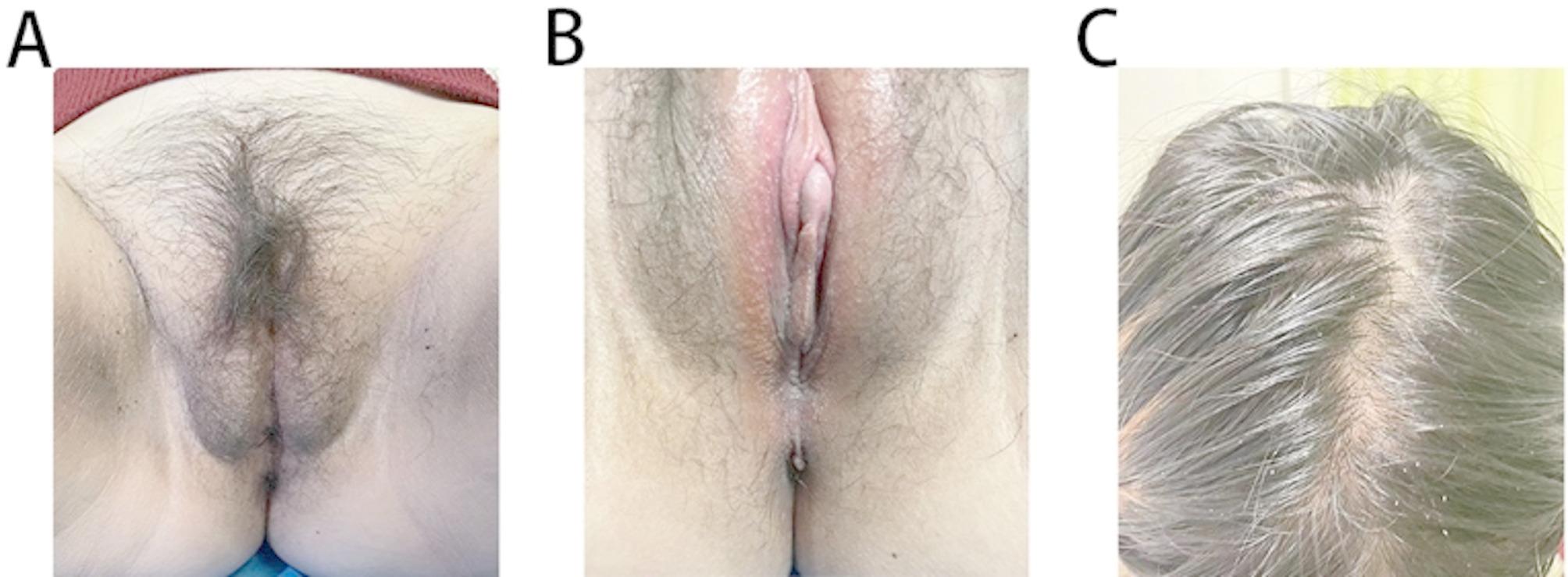
Clinical images. **A**: Acanthosis nigricans sign and hirsutism; **B**: Female external genitalia with enlarged clitoris; **C**: Greasy texture and seborrheic alopecia appearance of the hair

Physical examination revealed female external genitalia with an enlarged clitoris (clitoral index of 84 mm^2^). Hirsutism was reflected in the abnormally thickened terminal hair of her body in a male pattern (Ferriman–Gallwey score 12 points), primarily observed in androgen-sensitive anatomical areas [5]. Acanthosis nigricans signs were observed in the groin skinfold and inner thigh. Her hair had a greasy texture and exhibited signs of seborrheic alopecia [6] (Fig. 1A-C). She also experienced hoarseness of voice and obesity [7] (body mass index: 29.29 kg/m^2^).

### Laboratory examination

Laboratory examination revealed typical hyperandrogenism [8]. Test results during the initial outpatient visit in April 29, 2022 showed a total testosterone level of 3.60 ng/mL (normal range for Tanner 5 female < 0.40 ng/mL), free testosterone index (FAI) of 62.10 (normal range: 0.33–4.37), dehydroepiandrosterone sulfate (DHEAS) level of 464 µg/dL (normal range: 35–430 µg/dL), and androstenedione (AND) level of 8.72 ng/mL (normal range: 0.30–3.30 ng/mL). The test results before laparoscopic mass resection in February 9, 2023 were a testosterone level of 8.89 ng/mL, FAI of 29.00, DHEAS level of 416 µg/dL, and an AND level > 10.0 ng/mL (Table 2).

**Table 2:** Laboratory values before and after surgery

Date	Testosterone (< 0.40 ng/mL)	FAI (0.33–4.37)	DHEAS (35–430 µg/dL)	AND (0.30–3.30 ng/mL)
April 29, 2022	3.60	62.10	464	8.72
February 9, 2023 (The day before surgery)	8.89	29.00	416	> 10.0
February 11, 2023 (The first day after surgery)	0.39	1.3	189	1.41

Notes: The values ​​in brackets represent the normal range. FAI, free testosterone index; DHEAS, dehydroepiandrosterone sulfate; AND, androstenedione

### Imaging examination

**Fig. 2:**
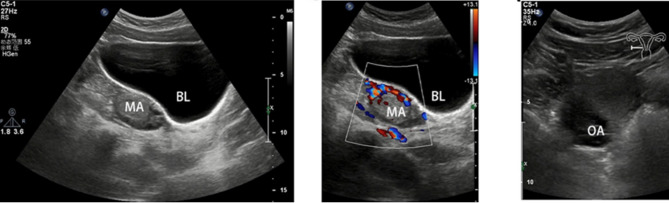
Ultrasound images. Mass (MA) on the left ovary (left and middle panels). Right ovary (OA) displayed characteristics of polycystic ovary syndrome (right panel). BL: Bladder

The patient underwent ultrasound imaging throughout the history of this illness. Before surgery, a slightly hyperechoic area was observed in the left ovary. Changes in the right ovary were also observed before and after surgery that possibly indicated PCOS (Table 3; Fig. 2). Color Doppler ultrasonography revealed a strong echogenic mass in the left ovary that was 2.4 cm at the initial visit and 2.6 cm after a 6-month follow-up in the outpatient department.

**Table 3:** Ultrasound findings

Date	Result
April 30, 2022	Slightly hyperechoic area observed in the left ovary, possibly a corpus luteum
May 9, 2022	Slightly hyperechoic area observed again in the left ovary, with a differential diagnosis of a teratoma or corpus luteum cyst
June 16, 2022	Slightly hyperechoic area observed again in the left ovary, with a differential diagnosis of a teratoma or corpus luteum cyst
November 21, 2022	Slightly hyperechoic area observed again in the left ovary; the right ovary showed changes possibly indicating PCOS
February 9, 2023 (preoperative ultrasound)	Slightly hyperechoic area observed again in the left ovary with a differential diagnosis of a teratoma
March 27, 2023 (postoperative ultrasound)	Left ovary appeared normal; right ovary showed changes indicative of PCOS
November 16, 2023 (postoperative follow-up ultrasound)	Right ovary showed changes indicative of PCOS

PCOS: Polycystic ovary syndrome

## Final diagnosis and treatment

The outpatient department diagnosed the patient with PCOS despite no alleviation of symptoms after a 5-month treatment with DIANE-35 [8].

**Fig. 3:**
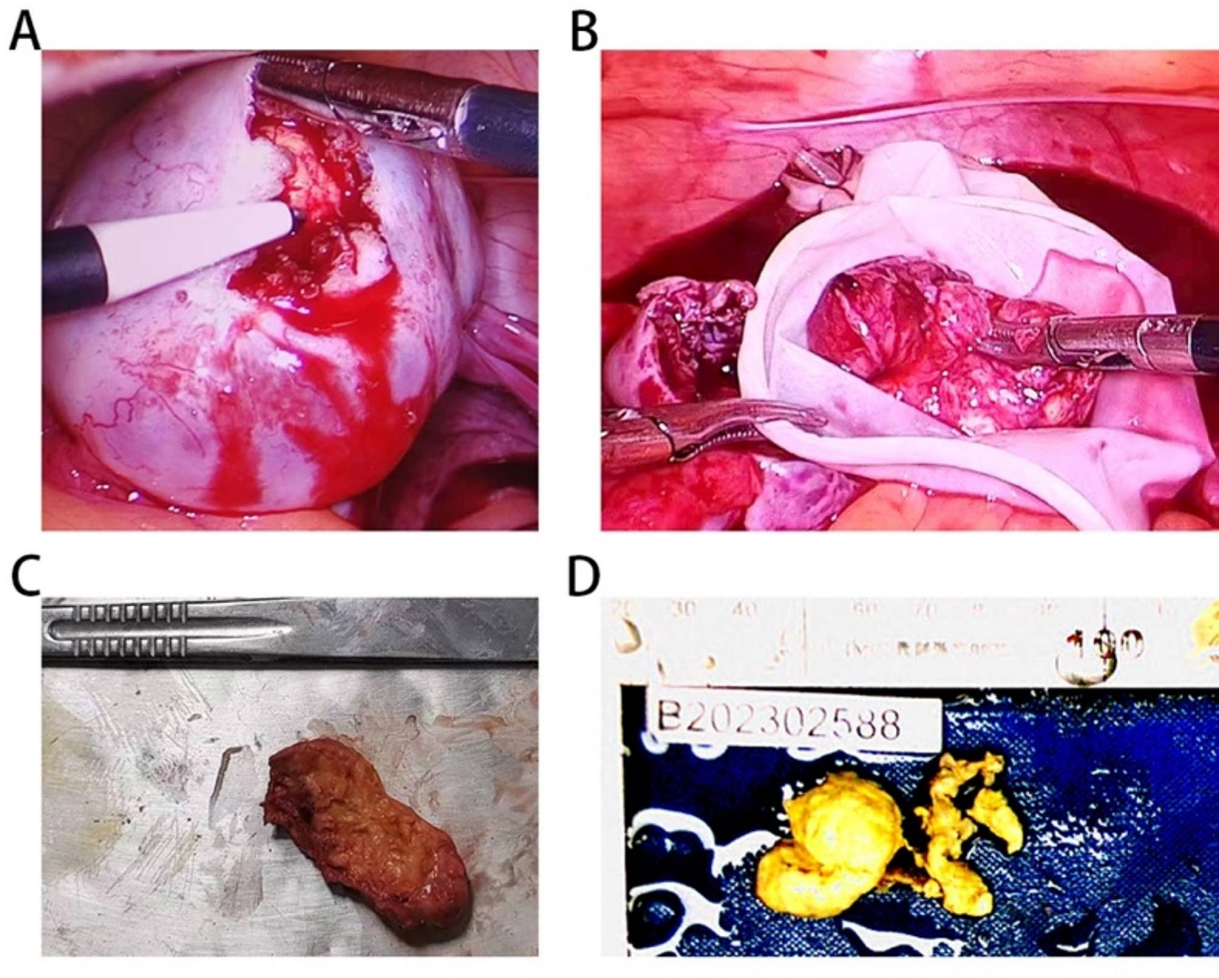
Surgical images. **A**: Incision of the enlarged ovary; **B**: Complete removal of the tumor from the ovary, which was placed into a surgical glove for extraction; **C**: Tumor appearance before formaldehyde fixation; **D**: Tumor appearance after formaldehyde fixation

**Fig. 4:**
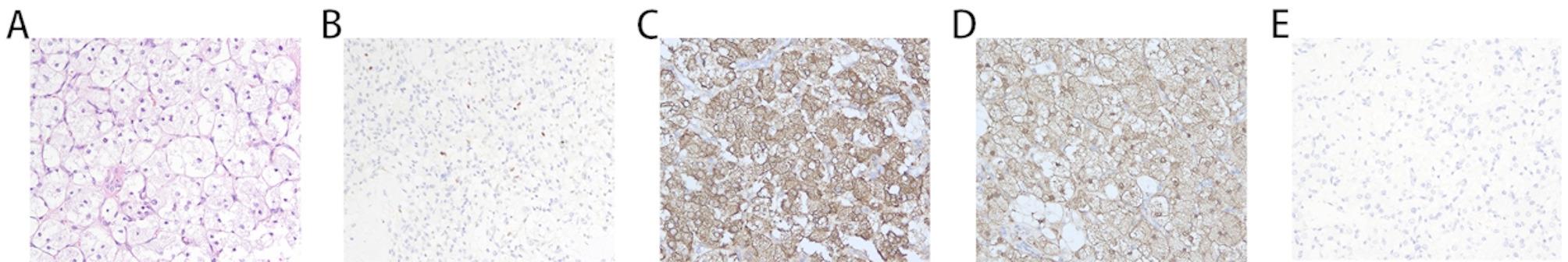
Pathological images. **A**: Hematoxylin-eosin staining; **B**: Ki67 staining; **C**: Immunohistochemistry of α-inhibin; **D**: Immunohistochemistry of calretinin; E: Ethidium monoamide staining. Magnification: × 200

On February 10, 2023 an exploratory laparoscopic surgery was performed to excise the affected ovarian cyst and to clarify the nature of the hyperechoic lesions in the ovaries and the reason for the poor treatment response for hyperandrogenism. Macroscopic observation revealed a yellow-brown adipose tissue-like tumor with a diameter of 3 cm and a clear boundary within the left ovary (Fig. 3A and D). Histopathological analysis revealed a nonspecific steroid cell tumor. Immunohistochemistry of the tumor revealed strong positivity for calretinin, α-inhibin, β-catenin, vimentin, P-CK, HNF1-B, ER, PR, and Ki67 and negativity for CD117, CD34, CA125, EMA, GATA3, FOXL2, S-100, and AR (Fig. 4A and E). These results confirmed the diagnosis of OSCT.

## Outcome and follow-up

On February 11, 2023, the first day post-surgery, the levels of testosterone and other androgenic hormones significantly decreased, confirming that the OSCT was the source of the hyperandrogenism [9]. The symptoms of hyperandrogenism improved after the surgery; specifically, testosterone levels decreased from 8.89 ng/mL to 0.39 ng/mL, DHEAS levels decreased from 416 µg/dL to 189 µg/dL, FAI decreased to 1.3, and AND levels decreased to 1.41 ng/mL (Table 2). During the post-surgical follow-up on April 3, 2023, the patient reported that no menstruation had occurred since the surgery. However, during a subsequent outpatient visit on November 15, 2023, the patient mentioned that menstruation had occurred in August, 2023.

## Literature review method

While OSCT and its synonyms, ovarian steroid cell tumor, ovary steroid cell tumor, ovarian steroid cell tumor, ovarian steroid cellular tumor, were used, PCOS and its synonyms, polycystic ovarian syndrome, polycystic ovary syndrome, polycystic ovarian syndromes, polycystic ovary syndromes were involved to the search target. These two topics were searched and joined with AND in Pubmed (pubmed.ncbi.nlm.nih.gov) on March 1, 2025. Other literature types were excluded except case report using the filter provided by Pubmed. Then we limited the search result using tittle/abstract filter provided by Pubmed to get pre-target case reports. All full text of pre-target papers were read and find out similar case reports which report clinical cases of OSCT-NOS misdiagnosed as PCOS. This search strategy was done by two co-authors independently.

## Literature review results

**Fig. 5:**
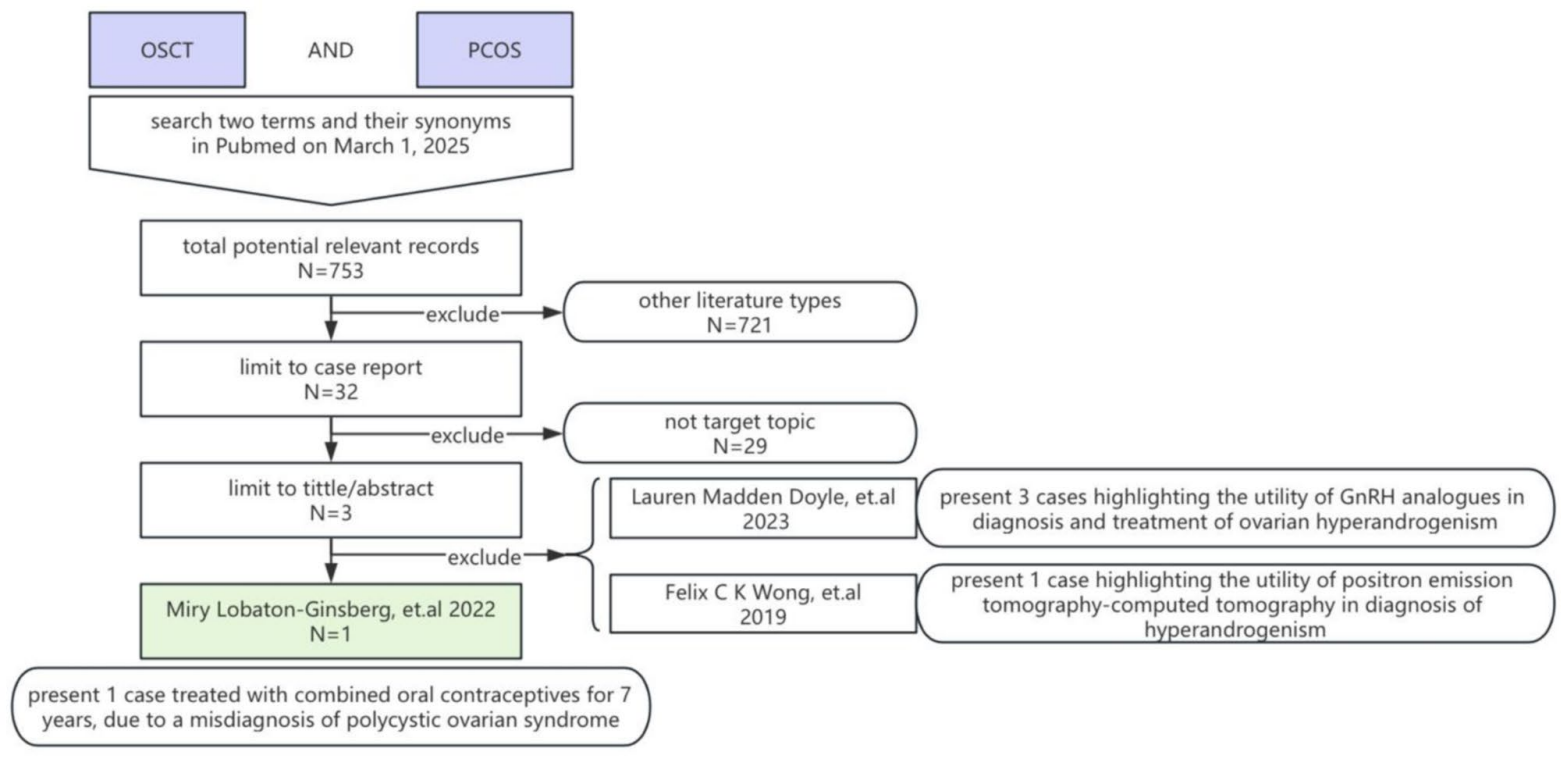
The search strategy and data selection flow diagram for literature review. OSCT: Ovarian steroid cell tumor; PCOS: Polycystic ovary syndrome; N: number of records

We do the review of literature as introduced in the previous section, and the same results were showed out as Fig. 5. As showed, only one case was similar with the clinical feature of the case reported by us (Fig. 5). They treated a 20-year-old Mexican woman with combined oral contraceptives from 13 years old, which lasted about 7 years, due to a misdiagnosis of PCOS. Biochemically, she had high serum total testosterone (7.0 ng/mL) and free testosterone levels (47 pg/mL), and a pelvic and transvaginal ultrasound followed by an abdominopelvic computed tomography (CT) scan demonstrated a right adnexal tumor. She underwent right salpingo-oophorectomy and the histopathological and immunochemistry exams confirmed the diagnosis of ovarian steroid cell tumors, not otherwise specified (OSCT-NOS). That patient was followed for a year after surgery and until then, her menses were regular and she had no recurrence of virilization signs [4].

## Discussion

Hormonal or biochemical hyperandrogenism is characterized by elevated levels of serum androgens [9]. Hyperandrogenism presents in females as significantly variable clinical symptoms. Importantly, it is possible to observe the physical manifestations of hyperandrogenism without biochemical hyperandrogenism [10]. These clinical manifestations include hirsutism, moderate-to-severe acne, irregularities in menstruation, virilization, and androgenic alopecia. Hirsutism is characterized by excessive growth of male-pattern terminal hair in females and affects up to 15% of the females. This prevalence may vary based on the population under study and the method employed to ascertain its presence [11]. However, the severity of hirsutism may not always reflect the circulating androgen concentration [12]. Excessive hair growth in females can also present as hypertrichosis. Therefore, it is necessary to distinguish hirsutism from hypertrichosis. Hypertrichosis typically has no pathological background and is observed in both male and females, independent of age, in terminal or vellus hair. This is an androgen-independent condition [13].

OSCT, not otherwise specified (OSCT-NOS) is a rare subtype of OSCT. OSCT-NOS, which accounts for almost two-thirds of all OSCT cases, primarily affects females in their 40s, although it can occasionally be detected before puberty [14]. OSCT shares numerous clinical characteristics with more prevalent conditions, such as PCOS. Both PCOS and OSCT may present with signs of hirsutism, irregularities in menstruation, moderate-to-severe acne, male pattern baldness, hoarseness of voice, and obesity [15]. In the current study, the patient presented with symptoms of progressive hirsutism since puberty, acanthosis nigricans, seborrheic alopecia, hoarseness of voice, and obesity. In addition, she experienced significant menstrual irregularities, such as prolonged periods of amenorrhea and irregular menstruation following withdrawal bleeding. She was initially misdiagnosed and treated for PCOS. Similar to this misdiagnosed girl, a young Mexican woman with OSCT-NOS had been treated with combined oral contraceptives from 13 years old, due to a misdiagnosis of PCOS [4]. The most important of all, PCOS is a diagnosis of exclusion and other potential disorders should be excluded.

Another differential diagnosis to considered is non-classical congenital adrenal hyperplasia (NCAH). It is a mild form of congenital adrenal hyperplasia (CAH), which is a group of autosomal recessive disorders linked to enzyme deficiencies in the cortisol and aldosterone synthesis pathway [16, 17] with an estimated global prevalence of 1 in 1000 births [18]. The symptoms of NCAH in adolescents also overlap with PCOS and include hirsutism, acne, irregular menstruation, and polycystic ovarian morphology (PCOM) on ultrasound (increased ovarian volume and/or number of secondary follicles) [17]. The primary diagnostic test for ruling out NCAH in patients with hyperandrogenism and menstrual irregularities is the measurement of 17-OH progesterone levels on days 3–5 of the menstrual cycle [19]. This differential diagnostic step is crucial for ensuring an accurate diagnosis. Considering the accurate test results need sample collected during the follicular phase of menstruation, and the surgery had been planned to done during her holiday, the 17-OH progesterone test was not provided to the girl to save medical expenses and meet her limited available holiday time. In the case reported before, 17α-hydroxy-progesterone (17-OHP) is 4.6 ng/mL (0.6–1.2 ng/mL) and she had a 17-OHP of 2.3 ng/mL after dexamethasone suppression test, which indicated a positive result and the false of the diagnosis of PCOS [4].

The overlap in clinical presentation underscores the diagnostic challenges when a female presents with hyperandrogenic symptoms. It is imperative to consider the potential differential diagnoses, including PCOS, NCAH, ovarian or adrenal tumors, hyperthecosis, and androgen-secreting neoplasms [20]. A large study involving 873 patients provided insights into the prevalence of conditions caused by excessive androgen levels. The authors reported that 0.2% of the patient population presented with androgen-secreting neoplasms, 0.6% were diagnosed with CAH, 1.6% were diagnosed with NCAH, 3.1% with HAIR-AN syndrome, 4.7% with idiopathic hirsutism, and 82.0% with PCOS [21]. The rarity of OSCTs often leads to delayed diagnosis, which can have critical implications on patient outcomes.

To prevent misdiagnosis, comprehensive laboratory and imaging tests are crucial. Hyperandrogenism is a key feature of PCOS, HAIR-AN syndrome and OSCT. Most OSCT patients have elevated preoperative androgen levels, usually more than 2 times of the reference value, similar to the current case. Our patient exhibited elevated FAI and increased DHEAS and AND levels, demonstrating a typical hyperandrogenism laboratory profile. Actually, while PCOS patients usually have only mildly elevated testosterone levels, usually less than 2 times of the reference value, PCOS and HAIR-AN syndrome also could show similar hyperandrogenism laboratory profile. The different is HAIR-AN syndrome patients should have insulin resistance (IR) and PCOS patients also could have IR, while simple OSCT women should not. And hyperandrogenism should be controlled by anti-androgen therapy in PCOS and HAIR-AN syndrome patient, while OSCT always lack of response to hormonal therapy. Moreover, after tumor removal, postoperative normalization of androgen levels occurs in most OSCT patients, which was also the experience of our patient, and this won’t happen in PCOS and HAIR-AN syndrome. The half-life of testosterone in the body is less than 100 min. According to the calculation of five half-lives reaching clearance (less than 5%), the patient’s testosterone level should return to normal within 8 h after the surgery. This highlights the surgical operation reversible nature of these hormonal imbalances in OSCT [15]. The rapid alleviation of symptoms within 2–3 months postoperatively is a valuable point to emphasize, as this can help differentiate between OSCT and PCOS [15]. As follow-up details of current case showed, the patient did experience amenorrhea for about 6 months post-surgery. And the expect menstruation resume time is 2–3 months after the normalization of hormonal parameters. The delayed return of menstruation may cause by her enormous academic pressure and immature HPO axis in adolescent age. Although the Rotterdam criteria clearly mentioned that exclude other causes is prerequisite for diagnosis of PCOS, the issue of miss diagnosis of OSCT may not receive sufficient attention from gynecologists. To illustrate this, we do review of literature on the topic. As showed, only one case was similar with the clinical feature of the case reported by us (Fig. 5). They treated a 20-year-old women with combined oral contraceptives from 13 years old, which lasted about 7 years, due to a misdiagnosis of PCOS [4]. More attention should be paid to the discrimination of such slow growth and small size of the mass on the ovary when combined with hyperandrogenism, especially mass fixed on one side of adnexa. That means when the patient lack of response to hormonal therapy, we should always consider add a clinical red flag to warrant further investigation for ovarian tumors in similar cases.

Recent research has identified spexin as a novel marker for evaluating ovarian dysfunction in PCOS-related infertility and it was reported to have a highly significant screening value and reliable diagnostic methods to differentiate PCOS cases versus control [22]. Unfortunately, this patient did not test this bio-marker. Utilizing markers will enhance diagnostic precision, particularly in cases where biochemical and clinical findings are inconsistent. Incorporating these updated markers into diagnostic protocols will help differentiate PCOS from other hyperandrogenism conditions. Patients with OSCT could receive timely surgery if there are markers in differentiating OSCT from PCOS. More basic and clinical studies are necessary certainly, to find out such novel markers.

While imaging techniques such as pelvic ultrasonography and MRI are valuable in diagnosing both OSCT and PCOS, there are key characteristics of each of these conditions. OSCT has different echogenicity from the surrounding ovarian tissues and low impedance values [23]. The 2023 updated International Evidence-based Guideline for the Assessment and Management of PCOS [24] reported the accuracy of different ovarian morphology parameters for PCOS diagnosis. The follicle number per ovary (FNPO) has a sensitivity and specificity of 70-94% and 70-100%, respectively (among 4975 adults). The follicle number per section has a sensitivity and specificity of 21-94% and 60-100%, respectively (among 2516 adults). FNPO is the most efficient ultrasonography for identifying PCOM in adults. Establishing a PCOM threshold in adults of FNPO ≥ 20 in at least one ovary should be considered. There are no definitive criteria for characterizing PCOM in adolescents. If an ultrasound is deemed necessary and suitable for the individual, transvaginal ultrasound is more precise than transabdominal ultrasound.

Adolescent females presenting with irregular menstruation and signs of hyperandrogenism may often be considered for a diagnosis of PCOS due to its high prevalence and overlapping clinical features with other conditions. However, this diagnosis should be made after a thorough evaluation, including a detailed medical history, clinical examination, and laboratory assessment as recommended by the 2023 international guidelines [23]. There are no definitive criteria to define PCOM in adolescents. Therefore, ultrasound is not recommended as a routine diagnostic step in this age group [23]. In cases where the diagnosis remains uncertain or there is an inadequate response to initial medical treatment, further imaging may be warranted to rule out other potential causes, such as ovarian or adrenal tumors. This targeted approach ensures that rare but significant conditions are not overlooked while adhering to current guidelines. On ultrasound, OSCT appears as a solid tumor with blood flow signals similar to those of a corpus luteum cyst that are not particularly abundant, making it difficult to distinguish especially when the tumor volume is small. In this case, the ovarian mass is only about 2 cm. Therefore, ultrasound can detect this mass, but its ability to distinguish the nature of the mass is limited. According to previous case reports, OSCT on CT and MRI presents a tumor with clear boundary and no necrosis or bleeding inside [4]. This suggests we perform CT or MRI to assist the diagnosis of OSCT.

Pathological characteristics of OSCT are also useful in confirming the correct diagnosis [24]. Histopathology remains the gold standard for a definitive OSCT-NOS diagnosis [25]. The majority of OSCT-NOS tumors are unilateral, well-circumscribed, and vary in size from 1.2 cm to 45.0 cm, with an average diameter of 6.5 cm [26]. Microscopically, an OSCT is comprised of large, polygonal cells with vacuolated cytoplasm and smaller cells with dense, eosinophilic, granular cytoplasm. The cells are often organized diffusely or in small nests within a vascular stroma. One distinguishing feature of OSCT-NOS is the absence of Reinke crystals, which differentiates OSCT from Leydig cell tumors. Additionally, Leydig cell tumors are generally found in hilar locations and frequently associated with Leydig cell hyperplasia [27]. Some OSCT-NOS also contain fibromatous components similar to those in thecomas, although these account for less than 10% of the tumor [28]. Immunohistochemically, as WHO Classification of Female Genital Tumors (5th edition) announced, OSCT are typically positive for sex cord-stromal markers, such as inhibin, calretinin, and melan-A, but they are usually negative for FOXL2. In this case, inhibin and calretinin were obvious positive expression, which were crucial for the definitive diagnosis to OSCT combined with the hormonal feature of the patient. IHC markers for specific diagnosis of OSCT-NOS need to be exploded in the future.

In our case, the patient’s diagnosis was confirmed through surgery. Surgical resection remains the primary treatment option. The decision for unilateral salpingo-oophorectomy or tumor removal is based on fertility desires, tumor stage, histopathology, and the presence of malignant features [29]. Our patient was treated surgically to remove the ovarian mass. Nonsurgical treatments had been employed for several years prior to surgery with no effect on the size of the mass, which ultimately met the surgical indications for intervention.

Ultimately, this case underscores the need to recognize that human errors and diagnostic challenges occur, particularly when common conditions like PCOS overlap with rarer diagnoses like OSCT. This case also highlights the importance of a thorough diagnostic process that considers a wide range of potential diagnoses, especially when initial treatments are ineffective. In previous case report [4] and current case, the most specific manifestations of OSCT were extremely elevated androgen levels with non-responsiveness to drug treatment, and combined with ovarian mass. This might serve as early diagnostic clue. We acknowledge that the conclusions drawn from the single case presented in this manuscript may seem limited in generalizability; however, other studies including a series of 63 cases [30], a report of 73 cases [26], and a case report of 3 cases [28] have provided further clinical insights into OSCT. Larger cohort studies are necessary certainly, to further substantiate and generalize our conclusions.

## Conclusion

OSCTs present a diagnostic challenge due to the rarity of this lesion and the overlapping clinical features with PCOS, a more common medical condition. A combination of clinical, laboratory, and imaging studies is essential for accurate diagnosis and timely management to ensure optimal outcomes in patients with OSCT.

## Data Availability

No datasets were generated or analysed during the current study.

## Declarations

### CARE checklist (2016) statement

The authors have read the CARE Checklist (2016), and the manuscript was prepared and revised according to the CARE Checklist (2016).

## Abbreviations

AND: Androstenedione
AR: Androgen Receptor
CAH: Classical adrenal hyperplasia
DHEAS: Dehydroepiandrosterone sulfate
EMA: Epithelial Membrane antigen
ER: Estrogen receptor
FAI: Free testosterone index
FNPO: Follicle number per ovary
FNPS: Follicle number per section
FOXL2: Forkhead Boc Protein L2
GATA3: GATA binding protein 3
HAIRAN: Hyperandrogenism, insulin Resistance and Acanthosis Nigricans
HNF1: B–Hepatocyte nuclear factor 1 beta
NCAH: Non–classical adrenal hyperplasia
OSCT: Ovarian Steroid Cell tumor
OSCT: NOS–Ovarian steroid cell tumor not otherwise specified
P: CK–Phosphorylated Cytokeratin
PCOM: Polycystic Ovarian Morphology
PCOS: Polycystic ovarian syndrome
PR: Progesterone receptor
